# The role of victimisation and sleep quality in self-harm and depression among sexual minority adolescents. A prospective cohort study

**DOI:** 10.1007/s00787-024-02444-4

**Published:** 2024-04-26

**Authors:** Alexandra Tepman, Mark Lawrence Wong

**Affiliations:** 1 Department of Psychology, University of Exeter, Exeter, UK; 2 Department of Social and Behavioural Sciences, City University of Hong Kong, Tat Chee Avenue, Kowloon, Hong Kong

**Keywords:** LGBT, Mental health disparities, Bullying, Gender stereotypes, Gender equality, Health and wellbeing

## Abstract

Sexual minority adolescents (SMA) have a disproportionately high prevalence of victimisation, self-harm, and depressed mood, relative to the general population. Yet, the contributing and mechanistic factors are unclear. We aim to explore the directional relationship between victimisation and self-harm and depressed mood, with poor sleep quality as a possible mediator. A secondary data analysis was conducted using a nationally representative birth cohort in the United Kingdom, where participants self-identified as sexual minority (*N* = 1922, aged 11–13, 67.1% female) and their parents completed questionnaires and interviews when the participants were aged 11, 14 and 17. Logistic and linear regression were used to test whether victimisation prospectively predicted self-harm and depressed mood with mediation analyses conducted to assess if sleep onset latency and nocturnal awakening mediated their relationships. After adjusting for demographic factors and baseline self-harm and depressed mood, victimisation at age 11 significantly predicted self-harm (*OR* = 1.40, *p* < .01) and depressed mood (*B* = 0.024, *SE* = 0.01, *p* < .05) at age 17. In the mediation analyses, frequent nocturnal awakening at age 14, but not sleep onset latency, significantly mediated the effect of victimisation at age 11 on self-harm (indirect effect *B* = 0.008, *SE* = 0.004, 95%CI = 0.001-0.017) and depressed mood (indirect effect *B* = 0.005, *SE =* 0.002 95%CI = 0.001-0.010) at age 17. Our findings supported that victimisation contributed to negative mental health among SMA. Poor sleep quality could be an indicator of maladjustment with victimisation, which further increased vulnerability to negative mental health. Victimisation and sleep quality could be important assessment targets in mental health campaign among sexual minority adolescents.

**Supplementary Information:**

The online version contains supplementary material available at 10.1007/s00787-024-02444-4.

## Introduction

Sexual minority adolescents (SMA) were noted to have increased risk of developing many negative health outcomes [1–2]. A recently published Just Like Us report [3] including 2,934 U.K. students aged 11–18, found that 42% of SMA experienced bullying, compared to 21% of non-SMA. The report also found SMA to have tripled the prevalence of self-injury behaviours, compared to non-SMA. While these evidence converged to indicate a higher prevalence of negative mental health outcomes among SMA, the underlying contributors and mechanistic factors were unclear and needed to be studied to facilitate the design of prevention and intervention for mental health problems of SMA.

### Victimisation, self-harm, and depressed mood

Victimisation was one of the main risk factors discussed in the literature for negative health outcomes among SMA. Victimisation was found to be about three times more common among SMA than non-SMA (see [4] for a meta-analytic review). Based on the Minority Stress Theory [5], sexual minority faced social stressors as a minority, e.g., prejudice, micro-aggression, homophobic victimisation, in addition to other stressors faced by non-sexual minority, increasing their vulnerability to mental health problems [6–7]. Therefore, conceivably, it might not be the sexual minority status per se, but the associated minority stressors contributing to negative mental health outcomes.

While existing research showed a significant association between victimisation and self-harm among the general adolescent population [8–9], relatively fewer studies focused on self-harm among SMA. Meta-analytical data [10] showed that 40.5% of the sexual minority population experienced self-harm, versus 24.4% of non-sexual minority. Another recent meta-analysis [4] stated that there was no conclusive indication regarding the causal relationship and mechanistic factors between victimisation and self-harm, since most existing studies were cross-sectional. Existing cross-sectional data showed that there were interrelations among victimisation and self-harm with other social factors, such as sense of connection with the social circle [11], internalised homophobia [12], as well as distress in hiding their sexual identity [13]. Among the limited studies with longitudinal designs, Burton and colleagues [6] found that sexual minority status at Time 1 prospectively predicted suicidal thoughts and behaviours at Time 2 (6 months later) and their association was mediated by sexual minority specific victimisation at Time 2. Such results suggested that victimisation was related to suicidal thoughts and behaviours, yet their directional relationship could be better inferred if these three factors were measured at different time-points [14]. Liu and Mustanski [15] assessed the prospective relationship between victimisation and self-harm among youths (aged 16–20) and found that victimisation due to sexual or gender minority status was one of the strongest predictors of self-harm apart from history of suicide. In addition, using a nationally representative USA youth sample (mean age = 15.9), Teasdale & Bradley-Engen [16] found that same-sex attraction and victimisation prospectively predicted suicidal attempts. Using a co-twin control design, O’Reilly and colleagues [2] recently reported that SMA were around 2 times more likely to self-harm or attempt suicide, relative to non-SMA, after adjusting for genetic and shared environmental factors. While these findings appeared to suggest victimisation as a predictor of self-harm, participants’ baseline self-harm behaviours were not controlled, limiting the inference of the individual contribution of victimisation.

Apart from self-harm, depressed mood was also found to be more prevalent among SMA than non-SMA [17]. For example, Burton and colleagues [6] included 197 students aged 14 to 19 and found that victimisation was associated with sexual minority status and depressed mood. la Roi and colleagues [18] found that among SMA (*N* = 153), victimisation mediated the relationship between sexual identity and later depression. However, in their study, self-reported victimisation was measured before sexual orientation was measured, which limited the inference on their temporal relationship. Another study using an England prospective cohort [19] found that victimisation mediated the difference in levels of depression between SMA (*N* = 187) and non-SMA (*N* = 3948). However, the sample did not include individuals born in other nations in the UK. In addition, all the aforementioned studies had less than 250 SMA participants [6, 18, 19] and studies with a larger number of SMA participants were needed to further verify the temporal association among victimisation and negative mental health outcomes.

### The role of sleep in mental health outcomes

Sleep problems were found to be more prevalent among SMA than non-SMA [20–23]. This could possibly be due to SMA’s increased exposure to victimisation, which heightened cognitive and emotional arousal, disrupting one’s sleep experience [24]. Research conducted among the general adolescent population showed that sleep problems prospectively predicted negative mood and self-harm [26–27], and sleep problems mediated the effect of victimisation on depressive symptoms [28, 29]. The link between sleep problems and negative mental health outcomes could be explained by sleep’s role in emotional regulation, which potentially affected individuals’ impulsivity and tendency towards self-injury behaviours (see [30] for a review). Despite the higher prevalence of victimisation and negative mental health in SMA, to the best knowledge of the authors, no existing study investigated the mediating role of sleep problems on victimisation and depressive symptoms and self-harm among the sexual minority population [31].

### The current study

This study investigated if victimisation prospectively predicted self-harm and depressed mood in SMA and whether poor sleep quality mediated the relationship, since sleep quality was (1) relatively less stigmatised, (2) easier to measure when compared to victimisation and (3) found to predict diverse negative mental health outcomes [20, 27]. We examined the difference in the types of victimisation experience and self-harm between SMA and non-SMA among our sample. We aimed to build on the limited existing longitudinal studies on victimisation and self-harm in SMA, e.g [6, 15, 16], by (1) measuring the predictor (victimisation), mediator (sleep quality) and outcome (self-harm and depressed mood) at three different time-points, allowing inference regarding temporal relationships [14]; (2) including the baseline measure of depressed mood and self-harm as covariates in the prospective analyses to more clearly gauge the contributions of victimisation and poor sleep quality; and (3) investigating the research questions using a nationally representative U.K. cohort which included participants across the four nations in the country. We hypothesized that the prevalence of victimisation and self-harm was higher for SMA than non-SMA. We also hypothesized that among SMA, victimisation at age 11 was a predictor for self-harm and depressed mood at age 17 after adjusting for the corresponding baseline measures. The relationship between victimisation and self-harm/depressed mood was hypothesized to be mediated by poor sleep quality at age 14.

## Methods

### Design

To investigate the research questions, we conducted a secondary data analysis using the Millennium Cohort Study (MCS), a nationally representative birth cohort of about 18,818 participants born in the years 2000–2002 across the nations in the UK [32]. Participants and their parents in the MCS provided informed consent before the data collection commenced and the current study received ethical approval for secondary data analysis. We extracted the data from the MCS when participants were at age 11, 14, and 17. The predictor was participants’ experience of age-11 victimisation, mediator was age-14 sleep quality and outcomes were age-17 self-harm and depressed mood (Sweep 7) (see Measures for details).

### Participants

In the MCS, 13,469 adolescents participated at age 11, with 11,872 at age 14, and 10,757 at age 17. Participants were included in this study if they have participated in these three time-points and completed the measures on victimisation and sexual identity (Supplementary Fig. 1). The final sample included 1922 SMA and 6900 non-SMA and their descriptive information was described in Table 1.

**Table 1:** Descriptive information of the sample

	*N*	Mean (SD) / %
Age	1922	11.15 (.32)
*Gender*		
Female Male	1228 603	67.1% 32.9%
*Sexual Identity*		
Mainly heterosexual/straight Bisexual Mainly gay or lesbian Completely gay or lesbian Other Do not know	978 593 83 132 124 12	50.9% 30.9% 4.3% 6.9% 6.5% .6%
*Ethnicity*		
White Mixed Indian, Pakistani and Bangladeshi Black or Black British Others	1584 90 76 31 27	87.6% 5.0% 4.2% 1.7% 1.5%
Weekly Family income (GBP-£)	1830	£458.4 (182.9)
*Bedtime during school nights*		
Before 9 pm 10–9:59 pm 10–10:59 pm 11 – midnight After midnight	75 452 693 447 147	4.1% 24.9% 38.2% 24.6% 8.1%
*Wake time during school days*		
Before 6 am 6–6:59 am 7–7:59 am 8– 8:59 am After 9 am	87 793 879 48 9	4.8% 43.7% 48.4% 2.6% .5%
*Sleep onset latency*		
- 0–15 minutes - 15–30 minutes - 31–45 minutes - 46–60 minutes - > 60 minutes	444 596 358 161 250	24.5% 32.9% 19.8% 8.9% 13.8%
*Nocturnal Awakening*		
- All of the time - Most of the time - A good bit of the time - Some of the time - A little of the time - None of the time	67 163 178 316 632 454	3.7% 9.0% 9.8% 17.5% 34.9% 25.1%

*Note* SD: Standard Deviation; GBP: British Pound Stirling (£)

### Measures

#### Sexual identity

Participants were asked to select one of the following options to best describe how they currently think of themselves, “Completely heterosexual/straight; Mainly heterosexual/straight; Bisexual; Mainly gay or lesbian; Completely gay or lesbian; Other; Do not know; not applicable; Prefer not to say”. Similar to other cohort studies, e.g [19, 33, 34], we coded those describing themselves as “Completely heterosexual/straight” as non-SMA, while those selecting “Not applicable or Prefer not to say” were excluded from the analysis with the rest, who were not exclusively heterosexual or SMA.

### Victimisation

At age 11, the adolescent participants reported the frequency of being hurt or picked on by other children with the following options, “most days”, “about once a week”, “about once a month”, “every few months”, “less often”, or “Never”. The responses were on a 1–6 scale where a smaller number represented more frequent experiences of victimisation. Participant’s parents were also asked if the participant has been picked on or bullied by other children and they responded “not true”, “somewhat true”, or “certainly true”. Given our primary interests on SMA’s self-reported depressed mood and self-harm behaviours, we used participants’ self-reported victimisation as the predictor in the analyses and reported descriptive data on self-reported and parent-reported victimisation (see Results). We also included the data of victimisation at age 14 for comparison analyses with non-SMA. At age 14, participants self-reported whether they had been exposed to a range of victimisation experiences, as yes/no binary items (See Table 2 for the types of victimisation experiences assessed). We coded victimisation at both ages as binary variables (Yes/No), where participants endorsing at least one victimisation experience were coded as having been victimised. For these items, there was an option of “Don’t know/Don’t wish to answer/No answer” and those selecting these responses were not included for analyses in this study.

**Table 2:** Victimisation in sexual minority adolescents and non-sexual minority adolescents

	Non-SMA		SMA			
	*N*	%	*N*	%	x^2^	*p*
**Age 11** – Self-reported Victimisation Yes No	6900 3851 3049	55.8% 44.2%	1922 1246 676	64.8% 35.2%	50.10	< 0.001
**Age 11** – Parent-reported Victimisation Yes No	6900 1561 5339	22.6% 77.4%	1922 554 1368	28.8% 71.2%	31.71	< 0.001
**Age 14** – Self-reported Victimisation Yes No	6386 2976 3410	46.6% 53.4%	1804 1068 736	59.2% 40.8%	89.34	< 0.001
- Insulted /threatened / shouted at Yes No	6384 2597 3787	40.7% 59.3%	1804 970 834	53.8% 46.2%	98.02	< 0.001
- Experienced physical violence Yes No	6384 1312 5072	20.6% 79.4%	1803 473 1330	26.2% 73.8%	26.63	< 0.001
- Been hit /used weapon against Yes No	6381 168 6213	2.6% 97.4%	1804 63 1741	3.5% 96.5%	3.79	0.052
- Had something stolen Yes No	6382 431 5951	6.8% 93.2%	1804 161 1643	8.9% 91.1%	9.88	0.002
- Been sexually assaulted Yes No	6382 132 6250	2.1% 97.9%	1802 102 1700	5.7% 94.3%	65.28	< 0.001

*Note* x^2^ – chi-square test; SMA: Sexual Minority Adolescents

### Self-harm

At age 17, self-harm was measured based on the Edinburgh Study of Youth Transitions [35], where participants completed 5 binary items (Yes/No) regarding whether they had self-harmed by different means, including cutting or stabbing, burning, bruising or pinching, taking an overdose of tablets or pulling out hair, in the past year (Supplementary Table 3). They were also asked to state if they had used any other ways to hurt themselves in the past year. Participants endorsing any self-harm behaviour were coded as having performed self-harm. At age 14, participants were asked if they had hurt themselves on purpose in any way and they provided a response of yes or no.

### Depressed mood

At age 17, depressed mood was measured by the following item in the Kessler 6 scale [36], “During the last 30 days, how often did you feel so depressed that nothing could cheer you up?” At age 14, depressed mood was measured by the short version of the Mood and Feeling Questionnaire [37], where there were 13 statements regarding participants’ recent mood and feeling, e.g. “I hated myself” or “I did everything wrong”. For each item, the response options were “not true”, “sometimes”, or “true”. The total score ranged from 13 to 39 and the scale had good internal consistency (α = 0.94). A higher score indicated higher level of depressive symptoms in both time-points.

### Sleep quality

Sleep quality was measured by participants’ sleep onset latency (SOL), and nocturnal awakening at age 14, where they reported how long they usually took to fall asleep and how frequency they awakened during sleep at the last 4 weeks (See Table 1 for details). Participants were also asked about their typical bedtime and wake time during school days for descriptive information regarding sleep patterns (Table 1).

### Demographic variables

Participants’ age, gender, ethnicity, and weekly family income were measured at age 14 (Table 1). The response options and details were reported in Supplementary Table 1.

### Data analysis plan

**Fig. 1:**
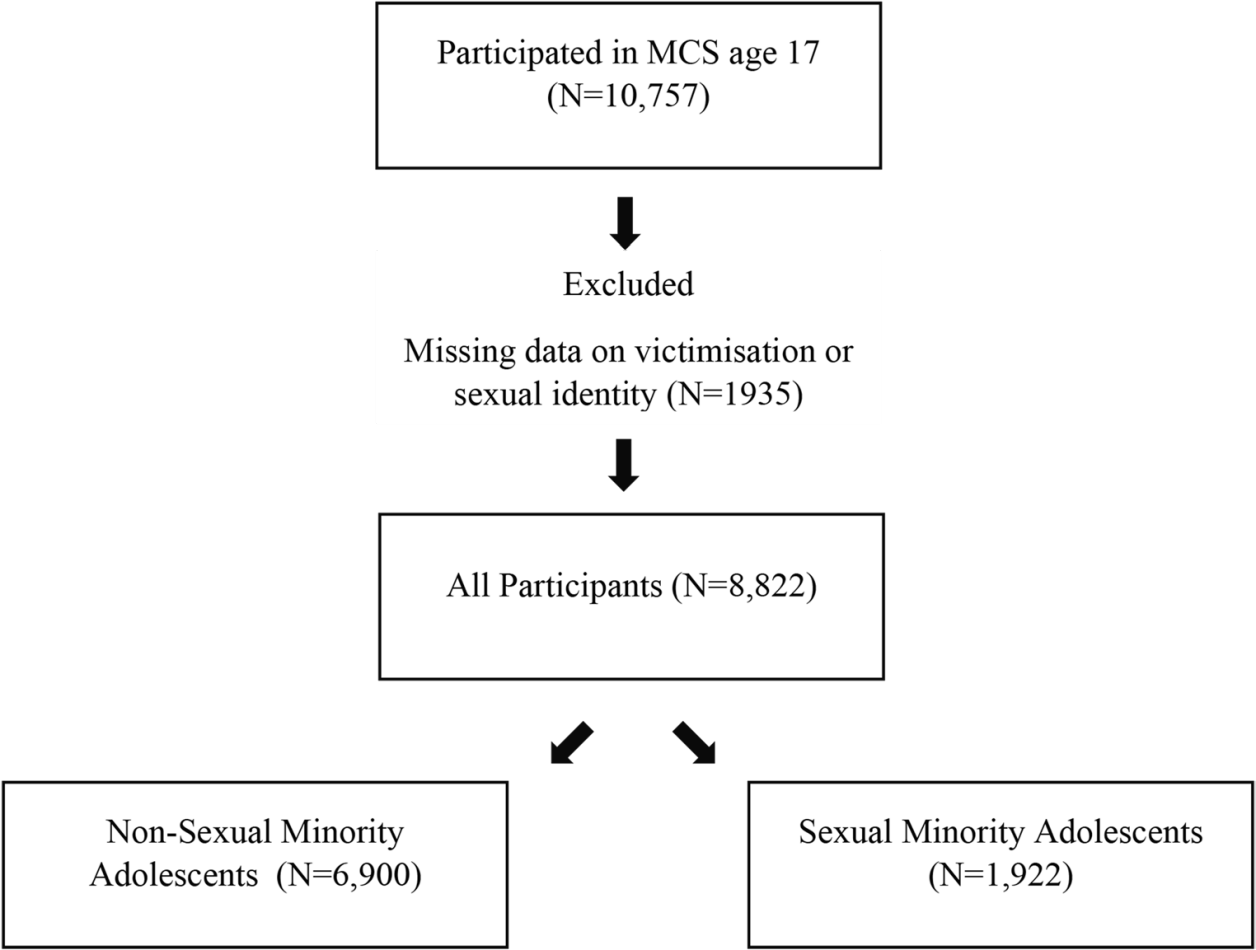
Participant flow chart

SPSS version 28 was used for all data analysis. Group differences were analysed using Chi-square or Mann-Whitney U test, given unequal variance. Correlational analyses were conducted to assess the cross-sectional relationship among victimisation, sleep quality, depressed mood, and self-harm at age 14. Binary logistic and linear regression were used to analyse the temporal relationship between victimisation, poor sleep quality with self-harm and depressed mood. The variables were tested for multi-collinearity and their variance inflation factor were lower than 10, indicating no significant concern of multi-collinearity. The regression analyses were conducted by entering demographic variables and baseline (age-14) of the outcome measure at step 1, victimisation at step 2 and SOL and nocturnal awakening at step 3. In view of multiple comparison and risk of inflated type-1 error, all *p*-values were adjusted following the Benjamini-Hochberg procedure with a false discovery rate (FDR) of 5%. Statistical significance was determined by an adjusted *p*-value, *p*_*fdr*_ <0.05. For the mediation hypotheses, a series of regression analyses were performed using the PROCESS Macro [38]. The regression analyses included Age-11 victimisation as the predictor, either age-14 SOL or nocturnal awakening as the mediator, and either age-17 self-harm or depressed mood as the outcome, with the corresponding measure at age 14 as the covariate (Fig. 1 and Fig. 2a and b). The model with self-harm as outcome was tested using logistic regression given that self-harm was coded as a binary (Yes/No) variable (Fig. 2a), while the model with depressed mood as outcome was tested using linear regression model given depressed mood was coded as a continuous variable (Fig. 2b). A bootstrap re-sample of 5000 was used to estimate the standard error. Significant mediation effect was inferred by a 95% confidence interval (95%CI) not containing 0.

**Fig. 2a:**
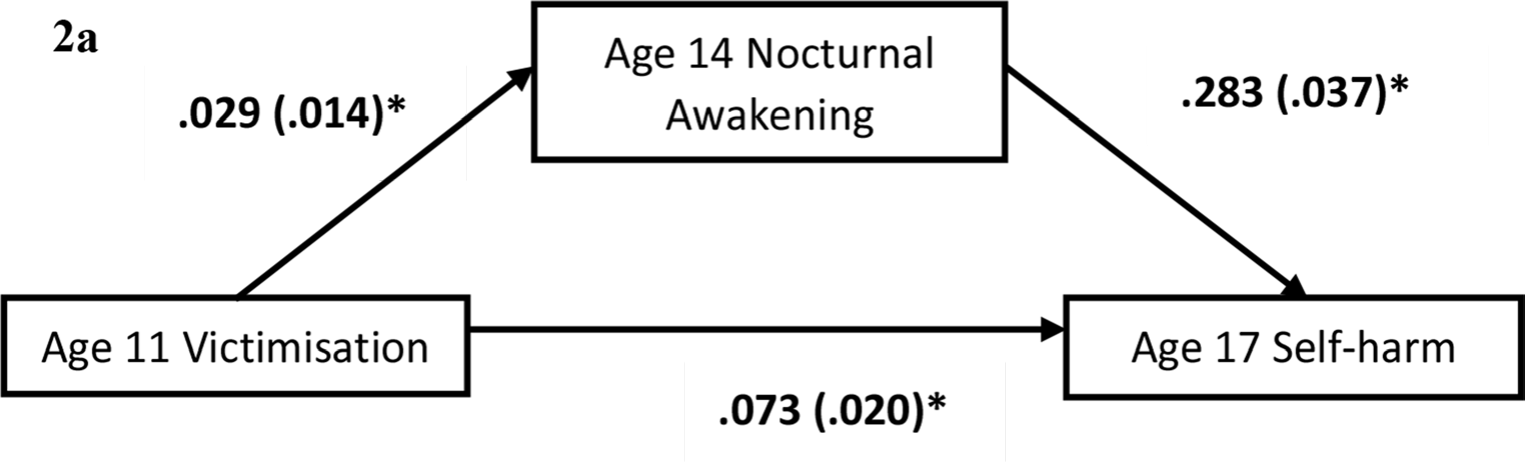
Nocturnal awakening’s mediation effect on victimisation and self-harm. *Note* Age 11 Victimisation has a significant indirect effect on Age 17 Self-harm through nocturnal awakening, B = 0.008, SE = 0.004, 95% CI: 0.001 − 0.017. Reported values are unstandardized regression coefficients and standard error. *95% CI do not include 0

**Fig. 2b:**
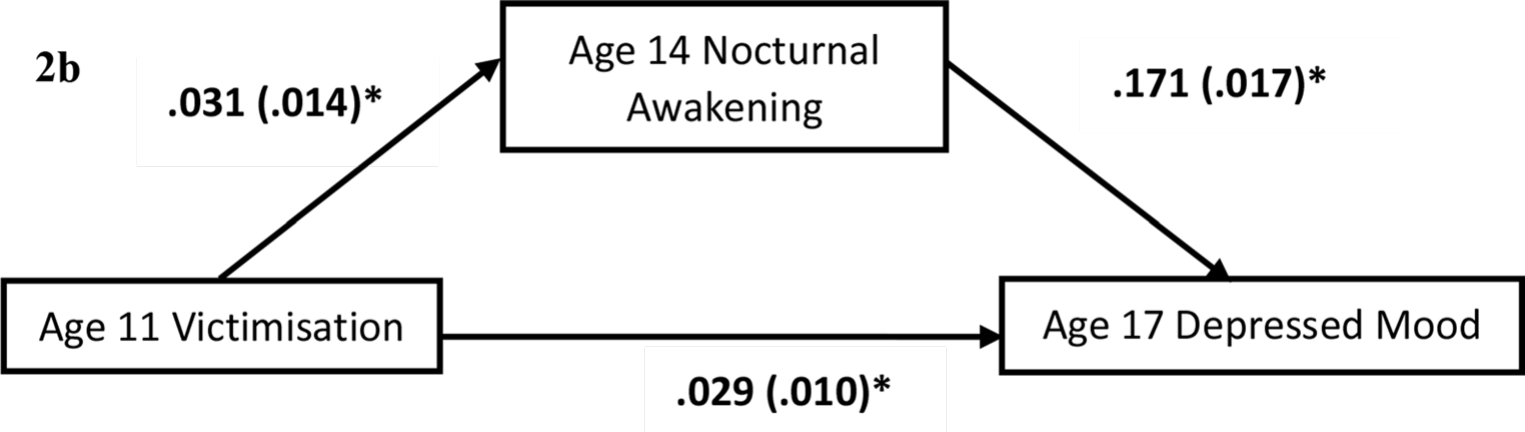
Mediation results for Depressed Mood. *Note* Age 11 Victimisation has a significant indirect effect on Age 17 Depressed Mood through nocturnal awakening, B = 0.005, SE = 0.002, 95% CI: 0.001 − 0.010. Reported values are unstandardized regression coefficients and standard error. * 95% CI do not include 0

## Results

Results from Mann-Whitney U test indicated that SMA had a higher level of depressive symptoms (*p*_*fdr*_<0.001), later bedtime (*p*_*fdr*_<0.001), later wake time (*p*_*fdr*_<0.001), longer SOL (*p*_*fdr*_<0.001), and more frequent nocturnal awakening (*p*_*fdr*_<0.001) than non-SMA. We conducted chi-square tests between SMA and non-SMA on victimisation and self-harm. The results showed that at age 11, both self-reported [*x*^2^(1) = 50.10, *p*_*fdr*_<0.001] and parent-reported victimisation [*x*^2^(1) = 31.71, *p*_*fdr*_<0.001], has a higher prevalence among SMA than non-SMA. In general, there was a higher rate of victimisation noted in self-reported data than parent-reported data for both SMA (64.8% versus 28.8%) and non-SMA (55.8 versus 22.6%) (Table 2). At age 14, SMA had a higher prevalence of all victimisation experiences, except for “having been hit/used weapon against”, than non-SMA. Of note, the prevalence of victimisation among SMA ranged from 3.5% (for being hit/used weapon against) to 53.8% (being insulted/threatened/shouted at) depending on types (Table 2). Regarding self-harm, at both time-points, SMA had a higher prevalence on all self-harm means than non-SMA. The prevalence of self-harm ranged from 6.6% (overdose of tablets) to 32.8% (self-bruising or pinching) among SMA, depending on the means (Supplementary Table 3).

### Cross-sectional relationship among Victimisation, sleep quality, self-harm, and depressed mood

Results from the Spearman Rank test showed significant cross-sectional associations among victimisation (all types considered) with poor sleep quality, self-harm, and depressed mood (*p*_*fdr*_<0.001). the individual correlation between each type of victimisation experience with sleep, self-harm and depressed mood were reported in Supplementary Table 2.

### Prospective analyses on victimisation, sleep quality, self-harm & depressed mood

Results from the logistic regression model showed that victimisation (*OR* = 1.40, 95%CI: 1.13–1.74, *p*_*fdr*_ =0.002), and nocturnal awakening (*OR* = 1.18, 95%CI: 1.09–1.28, *p*_*fdr*_<0.001) significantly predicted self-harm [*x*^2^(8) = 248.37, *p*_*fdr*_<0.001] after adjusting for the effects of demographic factors and self-harm at baseline (*OR* = 3.70, 95%CI: 2.89–4.75, *p*_*fdr*_<0.001) (Table 3). The results of mediation model showed that victimisation had a significant direct (*B* = 0.073, *SE* = 0.020, *OR* = 1.08, 95% CI = 0.033 − 0.11) and indirect effect on self-harm through nocturnal awakening (*B* = 0.008, *SE* = 0.004, *OR* = 1.008, 95% CI = 0.001 – 0.017) (Fig. 1). For the model with SOL as mediator, the indirect effect of victimisation on self-harm was not statistically significant (*B* = 0.001, *SE* = 0.003, *OR* = 1.001, 95% CI=-0.004 – 0.007) (Supplementary Fig. 2).

Regarding depressed mood, results from linear regression analysis showed that victimisation (*B* = 0.024, *SE* = 0.010, *p*_*fdr*_=0.016), SOL (*B*=-0.041, *SE* = 0.019, *p*_*fdr*_=0.035), and nocturnal awakening (*B* = 0.048, *SE* = 0.019, *p*_*fdr*_=0.012) significantly predicted depressed mood (*R*^2^ = 0.395, *F*_8,1770_=40.65, *p*_*fdr*_*<0.001*) after adjusting for the effect of demographic factors and depressed mood at baseline *(B* = 0.046, *SE* = 0.004, *p*_*fdr*_<0.001) (Table 3). In the mediation model with victimisation as the predictor, nocturnal awakening as the mediator, and depressed mood as the outcome, victimisation had a significantly direct (*B* = 0.034, *SE* = 0.010, 95% CI = 0.014 – 0.054) and indirect effect through nocturnal awakening (*B* = 0.005, *SE* = 0.002, 95% CI = 0.0006 – 0.0096) on depressed mood (Fig. 1). For the model with SOL as a mediator, the indirect effect of victimisation on depressed mood was not statistically significant (*B* = 0.001, *SE* = 0.002, 95% CI = − 0.003 – 0.005) (Supplementary Fig. 2).

**Table 3:** Effect of victimisation (age 11) on self-harm and depressed mood (age 17) after adjusting for the effects of demographic factors and other covariates

	Self-harm - age 17 *N* = 1922	Depressed Mood - age 17 *N* = 1778
	OR (95% CI)	B (SE)
Age – per year of age	0.949 (0.758-1.188)	0.019 (0.073)
Gender – female versus male	1.623 (1.301–2.024)***	− 0.152 (0.051)**
Weekly family income	1.000 (1.000-1.001)	0.001 (< 0.001)***
Ethnicity – non-white versus white	1.310 (0.956-1.797)	0.036 (0.072)
Self-harm age-14	3.703 (2.891–4.745)***	-
Depressed mood age-14	**-**	− 0.046 (0.004)***
Victimisation age-11	1.404 (1.134–1.739)**	0.024 (0.010)*
Sleep onset latency age-14	0.942 (0.868–1.024)	− 0.041 (0.019)*
Nocturnal Awakening age-14	1.182 (1.090–1.283)***	0.048 (0.019)*

*Note* *p < .05, **p < .01, ***p < .001; OR: Odd Ratios, 95% CI: 95% confidence Interval; B: unstandardized regression coefficient; SE: Standard Error

## Discussion

The primary aim of this study was to investigate the role of victimisation in the development of self-harm and depressed mood among SMA and the results indicated that victimisation prospectively predicted self-harm and depressed mood after adjusting for demographic factors and baseline self-harm and depressed mood. Frequent nocturnal awakening, as a measure of poor sleep quality, further mediated the effect of victimisation on self-harm and depressed mood. The interpretation of the findings is discussed in detail as follows,

### Victimisation and self-harm in SMA

In line with existing studies using samples from China [21], Sweden [38], Canada [39] and another UK birth cohort [34], our findings showed that SMA were generally more likely to experience victimisation, compared to non-SMA. In addition, while the prevalence of victimisation among SMA was estimated to be about three times more common than non-SMA [4], our data showed that the discrepancy on victimisation between SMA and non-SMA depended on the type of victimisation experience. For instance, while the prevalence of sexual assault was lower than most other victimisation experiences assessed, its prevalence among SMA (5.7%) was about 2–3 times higher than non-SMA (2.1%). However, the difference on other victimisation experiences, e.g., physical violence, between SMA (26.2%) and non-SMA (20.6%) might appear not as big as sexual assault, potentially due to its higher prevalence among both SMA and non-SMA. Also, similar to other studies [2, 34], our SMA sample had a greater prevalence of self-harm than non-SMA and we further found the extent of discrepancy depended on the means of self-harm, with greatest discrepancy noted among more life-threatening self-injury behaviours, e.g., overdose of tablets, self-cutting or stabbing. These findings collectively highlighted the importance of comprehensive assessment on victimisation types and self-harm means in understanding their prevalence among adolescents.

### Victimisation as an early predictor for later development of self-harm and depressed mood

Our findings showed that victimisation prospectively predicted negative mental health outcomes in SMA. Regarding self-harm, our results were consistent with existing studies conducted among older adolescents [15], where there was a prospective relationship between victimisation and self-harm in SMA populations. We also included more comprehensive measures of self-harm, as well as setting its baseline as covariate in the statistical analyses. The enhanced methodology quality provided further support for the predictive role of victimisation of self-harm and depressed mood among SMA. Our findings were also consistent with the result patterns found in the general adolescent population [40] that victimisation could prospectively predict later development of self-harm among SMA. For depressed mood, we found a prospective relationship between victimisation and depressed mood among the SMA population after adjusting for baseline depressed mood and other demographic factors. Our results were consistent with existing studies [18] which also found victimisation to predict depression among SMA, and we further verified the pattern of associations by using a nationally representative UK sample with a considerable number of SMA participants (*N* = 1922). These findings were in line with the Minority Stress Theory that the experience of victimisation and other minority stressors, contributed to the development of negative mental health outcomes among SMA.

### The role of poor sleep quality in victimisation, self-harm and depressed mood in SMA

Our results were in line with existing cross-sectional data between victimisation and sleep quality among the SMA [21] and prospective data among general adolescent population [40]. To the best knowledge of the authors, our findings were among the first to show that poor sleep quality, particularly frequent nocturnal awakening, prospectively predicted self-harm and depressed mood, after adjusting for baseline self-harm and depressed mood, among SMA. The significant relationship between poor sleep quality and negative mental health outcomes could be understood with existing cognitive neuroscience literatures which showed that sleep health was essential for optimal brain functioning in affect regulation [30]). While sleep quality was fairly direct to assess and a less stigmatised issue than victimisation, our findings potentially indicated that sleep quality and frequency of nocturnal awakening could be good assessment targets to help identify the vulnerable population at risk of developing negative mental health outcomes.

Inconsistent with our hypothesis, SOL did not significantly mediate the effect of victimisation on self-harm or depressed mood, which appeared inconsistent with some studies [41]. While longer SOL generally represented poorer sleep quality, among adolescents, short SOL could also reflect sleep debt because of insufficient sleep. Indeed, existing studies have found that adolescents with insufficient sleep had shorter SOL than those with sufficient sleep [42]. Future studies which investigate both sleep duration and SOL, using both subjective and objective data could further address the role of SOL in SMA’s wellbeing.

On the other hand, our data showed that nocturnal awakening prospectively predicted depressed mood and self-harm and mediated the effect of victimisation on them. Frequent nocturnal awakening conceivably represented a higher level of nocturnal arousal and difficulty to relax, which could be a behavioural manifestation of unsatisfactory adjustment to victimisation, ultimately increasing individual’s vulnerability to negative mental health outcomes. Though we had no measure regarding the reasons for nocturnal awakening, frequent nocturnal awakening could also be due to nightmare or insomnia which could be triggered by victimisation or unpleasant experience. Despite the different interpretations of the findings, our results added to a recent that sleep health had an important contributing to wellbeing among SMA [25].

### Limitations

Notwithstanding, this study had some limitations as follows. Firstly, we studied sexual minority as a homogenous group as other cohort studies [19, 33, 34], though a recent review stated that some specific sexual identities, e.g., bisexual, were at higher risk of developing negative mental health outcomes and were worth further investigation [23]. Secondly, the victimisation measure in this study did not capture the motivation/victim’s account of the victimisation experience, while some studies [6] showed that victimisation due to sexual orientation might have a stronger impact on mental health than due to other reasons. Thirdly, most of the measures were self-reported and future studies which include both self-reported and parent-reported and teacher-reported measures could more comprehensively address the role of victimisation on negative mental health outcomes. Fourthly, apart from victimisation and sleep problem, there were other unexplored factors, e.g. social connectedness, internalised homophobia/biphobia, which could be involved in the directional relationship among victimisation with self-harm and depressed mood [11–13].

## Conclusion

To conclude, while existing studies consistently reported a higher prevalence of depressed mood and self-harm among SMA when compared to non-SMA, this study showed that victimisation experience, as a minority stressor, prospectively predicted these negative mental health outcomes. We additionally found that frequent nocturnal awakening among SMA, was a potential indicator of later development of negative mental health in SMA. Intervention studies which target sleep problems and victimisation experience among SMA will be helpful to further verify the directional relationship among these factors. Also, further longitudinal, and experimental data are needed to identify early indicators, or protective factors against self-harm and depressed mood, since if left untreated, their impact on wellbeing could persist into early adulthood [32] or have long-reaching implications on healthy development.

## Electronic supplementary material

Below is the link to the electronic supplementary material.

Supplementary Material 1

Supplementary Material 2

## Data Availability

This is a secondary data analysis and the details in accessing the data can be referred to the UK data service, https://ukdataservice.ac.uk/.
