# Supplementary material for: The role of victimisation and sleep quality in self-harm and depression among sexual minority adolescents. A prospective cohort study
Source: Eur Child Adolesc Psychiatry. 2024 Apr 26;33(11):3993–4002. doi: 10.1007/s00787-024-02444-4 (PMC11588763; doi:10.1007/s00787-024-02444-4)
Supplement: Supplementary file 1 — Supplementary Material 1 [file 787_2024_2444_MOESM1_ESM.docx]

**Supplementary Table 1**

*Responses options and details of items assessing demographic variables*

| **Area** | **Question** | **Response options** |
| --- | --- | --- |
| **Gender – young person self-report** | Which of the following describes how you think of yourself? | 1 Male  2 Female  3 In another way [Please write in the box below]  4 Prefer not to say |
| **Ethnicity – parent report** | What is your ethnic group? | 1 White - English/Welsh/Scottish/Northern Irish/British  2 White - Irish  3 White - Gypsy or Irish Traveller  4 Any other White background (PLEASE SPECIFY)  5 Mixed/multiple ethnic groups - White and Black Caribbean  6 Mixed/multiple ethnic groups - White and Black African  7 Mixed/multiple ethnic groups - White and Asian  8 Any other mixed/multiple ethnic background (PLEASE SPECIFY)  9 Asian/Asian British – Indian  10 Asian/Asian British – Pakistani  11 Asian/Asian British – Bangladeshi  12 Asian/Asian British – Chinese  13 Any other Asian background (PLEASE SPECIFY)  14 Black/African/Caribbean/Black British - African  15 Black/African/Caribbean/Black British – Caribbean  16 Any other Black/African/Caribbean background (PLEASE SPECIFY)  17 Other ethnic group – Arab  18 Any other ethnic group (PLEASE SPECIFY) |
| **Weekly family income – parent report** | Could you please think about your take home income in the last 12 months. That is, the amount you personally took out of the business after all taxes and costs. About how much is this? | Input exact value. The income of all members living in the household is summed to form this variable. |

**Supplementary Table 2**

*Correlation between all main variables among sexual minority adolescents at age 14*

|  | 1 | 2 | 3 | 4 | 5 | 6 | 7 | 8 | 9 |
| --- | --- | --- | --- | --- | --- | --- | --- | --- | --- |
| 1. Insults, threats, shouted at | 1 |  |  |  |  |  |  |  |  |
| 1. Physical violence | .402*** | 1 |  |  |  |  |  |  |  |
| 1. Being hit /used weapon against | .146*** | .292*** | 1 |  |  |  |  |  |  |
| 1. Something stolen | .177*** | .233*** | .195*** | 1 |  |  |  |  |  |
| 1. Sexually assaulted | .145*** | .144*** | .033 | .075** | 1 |  |  |  |  |
| 1. Victimisation - all types included | .895*** | .495*** | .158*** | .260*** | .204*** | 1 |  |  |  |
| 1. Self-harm | .209*** | .145*** | .097*** | .100*** | .180*** | .190*** | 1 |  |  |
| 1. Depressed mood | .139*** | .111*** | .066** | .085*** | .028 | .150*** | 0.274*** | 1 |  |
| 1. Sleep onset latency | -.136*** | .-.074** | -.042 | -.093*** | -.053* | -.142*** | -.211*** | -.175*** | 1 |
| 1. Nocturnal awakening | .181*** | .111*** | .057* | .152*** | .088*** | .189*** | .234*** | .222*** | -.345*** |

Note: Spearman correlation used for all correlations *p<.05; **p<.01; ***p<.001

**Supplementary Figure 1**

*Sleep onset latency’s mediation effect on victimisation and self-harm*

Age 14 Sleep Onset Latency

**-.211 (.037)***

-.007 (.013)

Age 11 Victimisation

Age 17 Self-harm

**.078 (.020)***

*Note: Age 11 Victimisation has a non-significant indirect effect on Age 17 Self-harm through Sleep Onset Latency, B=.001, SE=.003, 95% CI: -.004 - .007. Reported values are unstandardized regression coefficients and standard error. * 95% CI do not include 0.*

**Supplementary Figure 2**

*Sleep onset latency’s mediation effect on Victimisation and Depressed Mood*

Age 14 Sleep Onset Latency

Age 11 Victimisation

-.007 (.013)

**-.140 (.019)***

**.032 (.011)***

Age 17 Depressed Mood

*Note: Age 11 Victimisation has a non-significant indirect effect on Age 17 Depressed Mood through Sleep Onset Latency, B=.001, SE=.002, 95% CI: -.003 - .005. Reported values are unstandardized regression coefficients and standard error. * 95% CI do not include 0.*
