# Supplementary material for: The role of victimisation and sleep quality in self-harm and depression among sexual minority adolescents. A prospective cohort study
Source: Eur Child Adolesc Psychiatry. 2024 Apr 26;33(11):3993–4002. doi: 10.1007/s00787-024-02444-4 (PMC11588763; doi:10.1007/s00787-024-02444-4)
Supplement: Supplementary file 2 — Supplementary Material 2 [file 787_2024_2444_MOESM2_ESM.docx]

**Table 3** *Self-harm in Sexual Minority Adolescents and Non-sexual Minority Adolescents*

|  | **Non-SMA** | | **SMA** | |  |
| --- | --- | --- | --- | --- | --- |
|  | **N** | **%** | **N** | **%** | **X^2^** |
| **Age 14** – Self-harm  Yes  No | 6377  746  5631 | 11.7  88.3 | 1792 487  1305 | 27.2  72.8 | 261.51*** |
| **Age 17** – Self-harm (All)  Yes  No | 6768  1255  5513 | 18.5  81.5 | 1875 919  956 | 49.0  51.0 | 724.07*** |
| - Self-cutting or stabbing   Yes  No | 6754 481  6273 | 7.1  92.9 | 1868 489  1379 | 26.2  73.8 | 532.19*** |
| - Burned yourself   Yes  No | 6760 205  6555 | 3.0  97.0 | 1866 183  1683 | 9.8  90.2 | 156.23*** |
| - Self-Bruising or pinching   Yes  No | 6756 696  6060 | 10.3  89.7 | 1867 612  1255 | 32.8  67.2 | 574.36*** |
| - Overdose of tablets   Yes  No | 6757 134  6623 | 2.0  98.0 | 1868 124  1744 | 6.6  93.4 | 109.28*** |
| - Hair pulling   Yes  No | 6382 431  5951 | 6.8  93.2 | 1804 161  1643 | 8.9  91.1 | 249.18*** |
| - Others   Yes  No | 6741 188  6553 | 2.8  97.2 | 1848 173  1675 | 9.4  90.6 | 155.61*** |
| - Purposeful self-harm to end life   Yes  No | 6766 366  6400 | 5.4  94.6 | 1869  276  1593 | 14.8  85.2 | 186.34*** |

*Note. X^2^ – chi-square test; SMA: Sexual Minority Adolescents. ***p_fdr_<.001*
